# Expression of pyruvate dehydrogenase kinase-1 in gastric cancer as a potential therapeutic target

**DOI:** 10.3892/ijo.2012.1687

**Published:** 2012-11-06

**Authors:** HOON HUR, YI XUAN, YOUNG BAE KIM, GWANG LEE, WOOYOUNG SHIM, JISOO YUN, IN-HYE HAM, SANG-UK HAN

**Affiliations:** 1 Department of Surgery,; 2 Institute for Gastric Cancer Mechanism,; 3 Department of Pathology, Ajou University School of Medicine,; 4 Department of Molecular Science and Technology, Ajou University College of Natural Sciences, Suwon, Gyeonggi-do 443-749, Republic of Korea

**Keywords:** gastric neoplasm, chemotherapy, pyruvate dehydrogenase, prognosis

## Abstract

In contrast to mitochondria in healthy cells, which utilize oxidative phosphorylation, malignant cells undergo elevated glycolysis for energy production using glucose. The objectives of this study were to evaluate whether the expression of various molecules, including pyruvate dehydrogenase kinase-1 (PDK-1), is involved in the altered glucose metabolism associated with gastric cancer prognosis and to assess the role of a therapeutic agent in targeting glucose metabolism in gastric cancer. Immunohistochemistry was performed on gastric cancer tissues obtained from 152 patients who underwent curative resection to assess the expression of hypoxia-inducible factor-1α (HIF-1α), glucose transporter-1 (GLUT-1), hexokinase-2 (HK-2) and PDK-1. In an *in vitro* analysis, the lactate production and glucose uptake levels, cellular viability and 5-fluorouracil (5-FU) responses were evaluated before and after treatment with dichloroacetate (DCA), a PDK-1 inhibitor, in the MKN45 and AGS gastric cancer cell lines and in the non-cancerous HEK293 cell line. GLUT-1 and PDK-1 expression was significantly associated with tumor progression, although only PDK-1 expression was an independent prognostic factor for patients who received 5-FU adjuvant treatment. There was no significant difference in cell viability between the HEK293 and gastric cancer cell lines following DCA treatment. However, DCA treatment reduced lactate production and increased responsiveness to 5-FU in MKN45 cells, which expressed high levels of PDK-1 in comparison to the other cell lines. Thus, PDK-1 may serve as a biomarker of poor prognosis in patients with gastric cancer. In addition, PDK-1 inhibitors such as DCA may be considered an additional treatment option for patients with PDK-1-expressing gastric cancers.

## Introduction

Gastric cancer is one of the most common types of solid tumors worldwide and is the second highest cause of cancer-related deaths despite recent decreases in its incidence and mortality rates (1). Despite curative resection treatments, patients with gastric cancer who are diagnosed with advanced disease display poor prognoses (2). Several clinical studies regarding the use of adjuvant cytotoxic or radiation therapies following curative resection for the treatment of advanced gastric cancer have demonstrated that these procedures may be performed to increase patient survival (3,4). However, patients with advanced-stage disease also demonstrate a high frequency of recurrence despite adjuvant treatment, and treatment outcomes can vary widely. Recent molecular cancer research advances have led to the development of new agents that target cancer-specific molecules, including those related to cancer progression or prognosis. In this study, we sought to discover new therapeutic targets with expression profiles that could be correlated with cancer progression and prognosis in patients with gastric cancer.

In contrast to healthy cells, which utilize oxidative phosphorylation as an energy source, malignant cells undergo increased rates of glycolysis for energy under hypoxic and non-hypoxic conditions (5). The measurement of this glycolytic metabolic change in cancer cells using 2-fluoro-2-deoxy-D-glucose positron emission tomography (FDG-PET) has become a widely accepted diagnostic tool for assessing various cancers including gastric cancer (6). In addition, FDG-PET has been shown to be useful for predicting the chemotherapeutic response (7,8). Therefore, aberrant glucose metabolism in cancers can be used as a selective target for cancer treatment. Several therapeutic agents targeting glycolysis have been reported to have significant cancer cell cytotoxicity in preclinical studies, and some of these therapeutic agents have advanced into clinical studies (9). However, the use of therapeutic agents targeting glycolysis for the treatment of gastric cancer has not been reported.

Several studies have reported that the expression of glucose transporter-1 (GLUT-1), which is involved in the glycolytic pathway, is significantly associated with disease prognosis in gastric carcinoma (10,11). However, many additional molecules, including pyruvate dehydrogenase kinase-1 (PDK-1), are involved in cancer cell-associated processes including active glycolysis, mitochondrial dysfunction and glucose uptake (5,12). To our knowledge, the use of various molecules associated with aberrant glucose metabolism as prognostic biomarkers has not been investigated in gastric cancer. Moreover, the therapeutic potential of agents targeting glycolysis for the treatment of gastric cancer has not been evaluated. Thus, the objectives of this study were to evaluate whether the expression of various molecules involved in glycolysis could be correlated with the prognosis of patients with gastric cancer and to assess the effects of treatment with a therapeutic agent targeting glucose metabolism in gastric cancer cell lines.

## Materials and methods

### Patients and tissue microarray

This study was approved by the Ajou University Hospital Institutional Review Board (AJOU-MED-KSP-10-375). Samples from 152 patients, who were diagnosed with gastric adenocarcinoma and underwent curative gastrectomy and proper lymphadenectomy between September 2006 and April 2007, were collected in paraffin blocks and used for a tissue array. The pathological stage of these tissue blocks after resection was investigated using the International Union against Cancer (6th edition) classification criteria. All of the patients enrolled in this study were reassessed for the recurrence of gastric cancer or death every 3–6 months using computed tomography, tumor marker expression and physical examination. Patients who were pathologically diagnosed with stage II, III or IV cancer were recommended to receive adjuvant chemotherapy consisting of 5-fluorouracil (5-FU) for approximately 1 year beginning 4–6 weeks after surgery.

Hematoxylin- and eosin-stained slides from the primary tumors of the 152 patients were reviewed by a pathologist (Y.B. Kim). Two formalin-fixed, paraffin-embedded cores (1 mm in diameter) were removed from the central region of the primary tumor specimens and arranged into tissue microarray (TMA) blocks.

### Immunohistochemical staining

**Figure 1.**
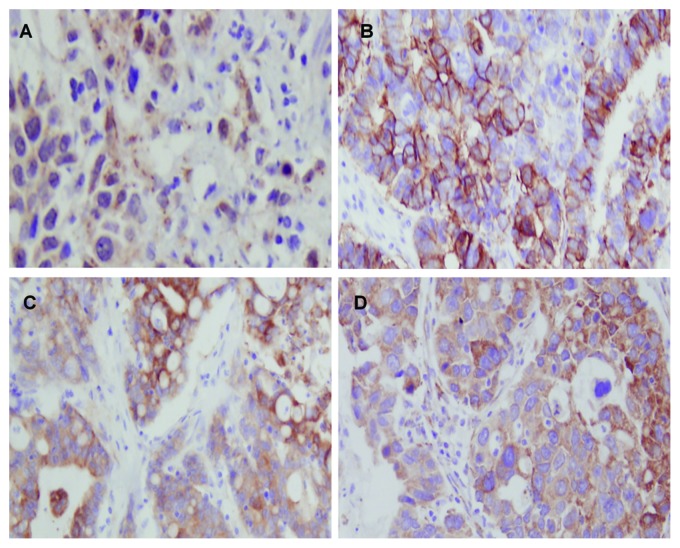
Immunoreactivity in immunostained gastric carcinoma samples. (A) Hypoxia-inducible factor-1α staining was localized to the cancer cell nuclei. (B) Glucose transporter-1 staining was observed at the cellular membrane. (C) Hexokinase-2 and (D) pyruvate dehydrogenase kinase-1 staining was localized to the cytoplasm (×400).

Briefly, 4-mm-thick sections were cut from the TMA blocks and deparaffinized. After immersion and blockade of endogenous peroxidase activity, immunohistochemical staining was performed using antibodies for hypoxia-inducible factor-1α (HIF-1α) (1:50 dilution; Thermo Fisher Scientific, Fremont, CA), GLUT-1 (1:200 dilution; Thermo Fisher Scientific), hexokinase-2 (HK-2) (1:50 dilution; Cell Signaling Inc., Danvers, MA, USA) and PDK-1 (1:400 dilution; Santa Cruz Biotech, Santa Cruz, CA, USA). The HIF-1α and GLUT-1 expression patterns were determined based on the nuclear, cytoplasmic (HIF-1α) and membrane (GLUT-1) staining. To measure the HK-2 and PDK-1 expression, the staining results were scored according to the number of tumor cells with positive expression. The proportion of tumor cells demonstrating positive staining was semi-quantitatively evaluated using the following scoring system: negative staining (<5% positive staining); 1+ staining (5–30%); 2+ staining (30–60%); and 3+ staining (>60%). A 2+ or 3+ expression level was considered positive expression. The expression of all molecules was determined if both regions were positively stained. All of the staining results were evaluated by two pathologists who were blinded to the clinical outcomes (Fig. 1).

### Cell lines and chemotherapeutic compound

The non-cancerous kidney cell line HEK293 and the gastric carcinoma cell lines AGS, MKN45, SNU-216, SNU-484, SNU-601 and SNU-638 were purchased from the Korea Cell Line Bank (Seoul, Korea). These cell lines were maintained in RPMI-1640 (Invitrogen Corp., Carlsbad, CA, USA) containing 10% fetal bovine serum (Equitech-Bio, Ingram, TX, USA), 100 U/ml penicillin G and 100 *μ*g/ml streptomycin (Invitrogen). The cells were incubated at 37°C in a humidified atmosphere containing 20% O_2_ and 5% CO_2_. 5-FU and dichloroacetate (DCA) were purchased from Sigma-Aldrich (St. Louis, MO, USA) and dissolved in deionized water to create 1 mol/l working solutions, which were filtered, sterilized and subsequently diluted in growth medium prior to treatment.

### Western blotting for PDK-1

Whole-cell lysates from cultured cells were lysed in a protein extraction solution (Intron Biotech., Sungnam, Korea). The lysates were centrifuged at 13,000 rpm for 15 min at 4°C to remove cellular debris. The protein concentrations were determined using the Bradford assay (Bio-Rad Laboratories, Hercules, CA, USA). In total, 25 *μ*g of protein were separated by SDS-polyacrylamide gel electrophoresis and transferred to a nitrocellulose membrane. After blocking and incubation, the primary antibodies used for western blot analysis, including anti-PDK-1 (1:1,000 dilution; Stressgen, Victoria, Canada) and anti-β-actin (1:10,000 dilution; Abcam, Cambridge, MA, USA), were applied. After incubating with the corresponding secondary antibodies, the signals were developed using an Amersham™ ECL Plus Western Blotting Detection System (GE Healthcare, Waukesha, WI, USA).

### Cytotoxicity following dichloroacetate and 5-FU treatment

HEK293, AGS and MKN45 gastric cancer cells were seeded at 1×10^5^ cells per well in 96-well plates, treated with different DCA concentrations (0, 10, 20, 30, 40 and 50 mM) and incubated for 24 h. Cell viability was measured using the novel tetrazolium compound 3-(4,5-dimethylthiazol-2-yl)-5-(3-carboxymethoxyphenyl)-2-(4-sulfophenyl)-2H-tetrazolium, inner salt [MTS(a)] assay kit (Promega, Madison, WI, USA). Next, 20 *μ*l of the MTS solution was added per well, the cells were incubated for 4 h, and the absorbance was measured by spectrophotometry at 490 nm. Three independent experiments were performed in triplicate.

To evaluate the synergic effects of DCA and 5-FU treatment, cells were treated with 5-FU (0–1,600 μM) alone or in combination with DCA (20 mM) and incubated for 24 h. The cell viability was measured using the same method described above.

### Glucose uptake assay

The glucose uptake assay was performed as previously described with modifications (13). Briefly, cells were seeded at 1×10^6^ cells per well in a 6-well culture plate and treated with DCA as described for the cytotoxicity assay. Following 24-h DCA treatment, the supernatant was discarded, and the cells were washed in a glucose-free medium. The glucose uptake level (without/with DCA treatment) was measured for each cell line using the Amplex Red Glucose/Glucose Oxidase Assay Kit (Invitrogen), according to the manufacturer’s instructions.

### Lactate production assay

After the cells were treated, the culture medium was removed and placed in another plate, and the cell number was calculated. Lactate assay components (Abcam) were then added to the removed culture medium, and the lactate levels were measured by spectrophotometry at 450 nm. The lactate concentration was normalized to the sample cell number.

### Statistical analysis

Statistical analysis was performed using the SPSS software, version 13.0. Correlations between the expression of each molecule and the clinicopathologic factors were evaluated using the χ^2^ test. Univariate analysis for the disease-free and overall survival rates and survival curve generation was performed using the log-rank test and the Kaplan-Meier method, respectively. A Cox proportional hazards model was used for the multivariate analysis to determine the prognosis-predicting factors. The difference in the mean values between two continuous variables was evaluated using Student’s t-test. Comparisons involving three variables were analyzed using a one-way ANOVA with the Scheffe *post hoc* comparison.

## Results

### Patient characteristics

The mean age of the patients was 55.8±13.6 years, and there were more male patients (63.2%) than female patients. Adjuvant chemotherapy was provided for 97 patients; of these patients, single-agent 5-FU was orally administered to 78 patients. The other patient characteristics are listed in Table I.

### Expression of molecules related to glucose metabolism in human tissues

Positive HIF-1α staining was observed in 47 out of the 152 patients (30.1%), and the staining was evident in the nuclei of the cancer cells. GLUT-1-positive staining was observed in 36 patients (23.6%), and the staining was localized to cellular membranes. Positive HK-2 and PDK-1 staining was detected in 7 (4.1%) and 19 (12.5%) patients, respectively, and this staining was evident in the cytoplasm. Positive staining for HIF-1α was significantly correlated with positive PDK-1 expression (p=0.029) (Table II).

The relationship between the clinicopathologic factors and the IHC results are listed in Table III. Positive GLUT-1 staining was significantly associated with tumor invasion (p=0.011) and lymph node metastasis (p=0.011). The histological classification results regarding the signet rings or mucinous cells were significantly different (p=0.042) from the other pathologic findings for GLUT-1 staining. For HK-2 expression, the staining results were not correlated with any of the clinicopathological features. However, PDK-1 staining significantly correlated with tumor invasion (p=0.020), the presence of a positive metastatic lymph node (p=0.040) and larger tumor size (p=0.006).

**Figure 2.**
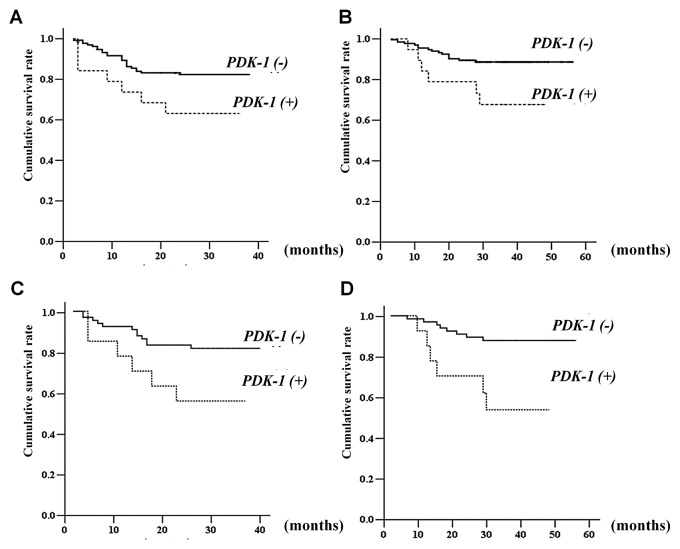
(A) Kaplan-Meier estimates of the disease-free and (B) overall survival rates for all patients enrolled in this study according to the expression of pyruvate dehydrogenase kinase-1. (C) The disease-free and (D) overall survival rates for 78 patients who were administered single-agent 5-fluorouracil adjuvant treatment.

The disease-free and overall survival rates were significantly associated with tumor size, the depth of invasion and lymph node metastasis (Table IV). With regards to the expression of these molecules, only PDK-1 expression was significantly correlated with the disease-free and overall survival rates (Fig. 2A and B). In 78 patients who were administered single-agent 5-FU as adjuvant treatment (Table V), patients with PDK-1 expression also demonstrated reduced disease-free and overall survival rates (Fig. 2C and D).

In the multivariate analysis, PDK-1 expression was one of the predictive factors for the overall survival rate (OR 2.890; 95% CI 1.108–7.536). For patients treated with single-agent 5-FU as adjuvant treatment, PDK-1 expression was one of the prognostic factors for early recurrence (OR 3.709; 95% CI 1.377–9,991) and short survival time (OR 5.132; 95% CI 1.699–15.503) (Table VI).

### Expression of PDK-1 and the cell line metabolic status

**Figure 3.**
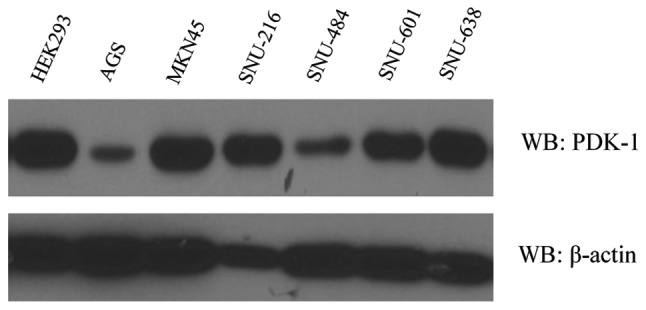
Seven gastric cancer cell lines and the HEK293 cell line were screened for their pyruvate dehydrogenase kinase-1 expression levels by western blot (WB) analysis.

As demonstrated by western blotting, PDK-1 was expressed in all of the gastric cancer cell lines as well as the HEK293 cells (Fig. 3). The PDK-1 expression level was lower in the AGS cell line in comparison to the other gastric cancer cell lines and the HEK293 cells.

**Figure 4.**
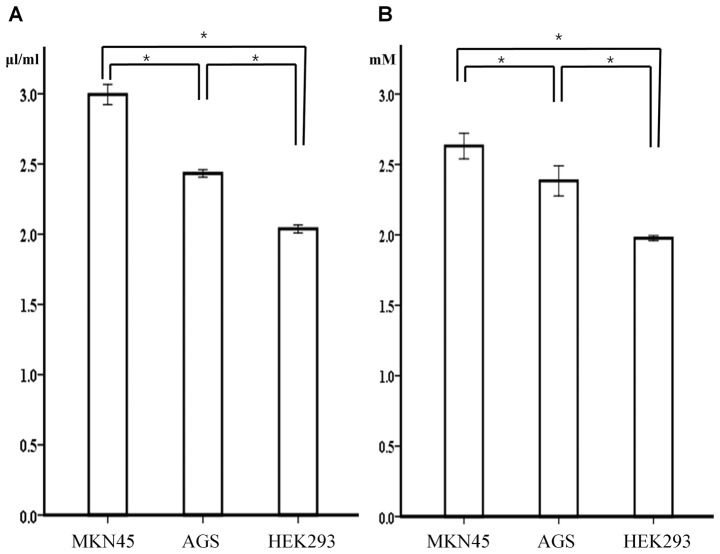
The level of (A) glucose uptake and (B) lactate production in the MKN45, AGS and HEK293 cell lines. The mean level of glucose uptake and lactate production in these cell lines was significant when evaluated using a one-way ANOVA with the Scheffe *post hoc* comparison (^*^p<0.001). The error bars indicate the standard deviation.

Of the gastric cancer cell lines, the glucose uptake and lactate production levels were significantly higher in the MKN45 cell line, which expressed a higher PDK-1 level in comparison to the AGS cell line, which expressed a lower level of PDK-1 (p<0.001). In addition, the non-cancerous HEK293 cells demonstrated a significantly lower level of glucose uptake and lactate production despite a higher level of PDK-1 expression when compared to MKN45 and AGS cells (Fig. 4).

### Effects of DCA and 5-FU on metabolism and viability

**Figure 5.**
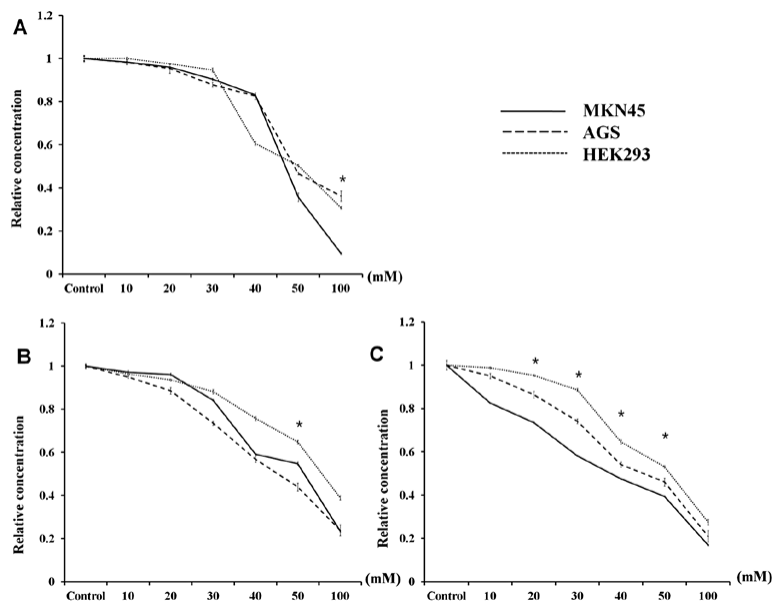
The characteristics of the response to dichloroacetate (DCA) treatment in the MKN45, AGS and HEK293 cell lines. (A) The cellular viability was measured using a proliferation assay, and the changes in the level of (B) glucose uptake and (C) lactate production following DCA treatment in the MKN45, AGS and HEK293 cell lines are shown. The mean level of the relative concentration in the three cell lines was evaluated using a one-way ANOVA with the Scheffe *post hoc* comparison (^*^p<0.001). The error bars indicate the standard deviation.

Following DCA treatment, the viability of each cell line was similar, with the exception of the highest DCA concentration (100 mM) (Fig. 5A). In addition, glucose uptake demonstrated a pattern similar to that observed for cell viability, although the change was the least pronounced in the non-cancerous cell line HEK293 (Fig. 5B). However, the lactate production in all three cell lines was significantly different when the cells were treated with 20–50 mM DCA (p<0.001). In particular, the lactate production in MKN45 cells, which demonstrated the highest level of PDK-1 expression by western blotting, demonstrated the largest decline after DCA treatment. In contrast, the effect of DCA on the decrease in lactate production was lowest in HEK293 cells (Fig. 5C).

**Figure 6.**
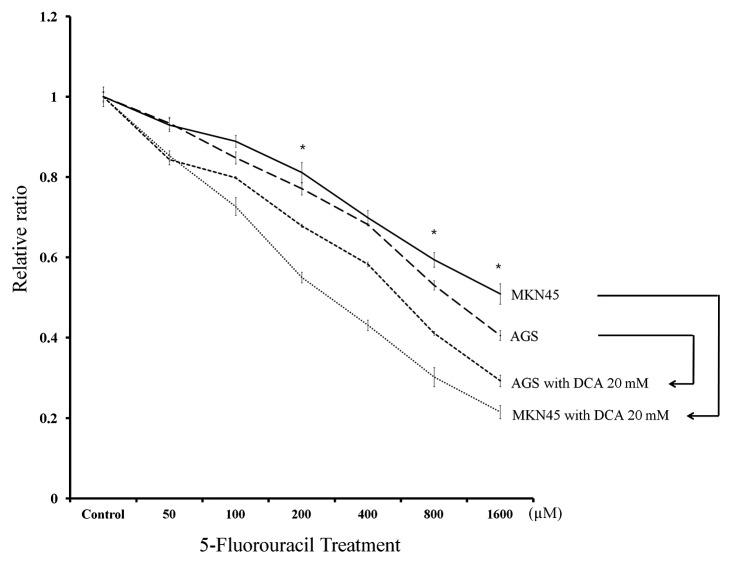
MKN45 and AGS cells were treated with 5-fluorouracil (5-FU) alone or in combination with 20 mM dichloroacetate (DCA). The responsiveness to 5-FU was lower in MKN45 cells, and the synergic effectiveness of DCA was higher in these cells compared to the AGS cells. The mean level of the relative concentration in MKN45 and AGS cells was compared using an independent t-test (^*^p<0.05). The error bars indicate the standard deviation.

We next evaluated the responsiveness of the cancer cell lines to 5-FU treatment alone or in combination with 20 mM DCA (Fig. 6). MKN45 cells demonstrated decreased responsiveness to 5-FU treatment compared to AGS cells following treatment with 200, 800 and 1,000 μM 5-FU (p<0.001). However, the synergic effect of DCA treatment was more pronounced in MKN45 cells. The mean relative ratio of cell viability following 1,000 μM 5-FU plus DCA treatment was reduced to 42.3% in MKN45 cells compared to 72.1% in AGS cells.

## Discussion

As suggested by Warburg, the level of aerobic glycolysis is a significant phenotype representing the metabolic changes that occur in solid tumors (14). Warburg reported that most of the cellular energy required for tumor survival and proliferation is produced by glycolysis, whereas very little mitochondrial energy production occurs in cancer cells. Due to the altered metabolism of cancer cells, the hypoxic or acidic tumor microenvironment has been evaluated in previous studies (15). In hypoxic conditions, solid tumors can adapt their metabolic pathways to regulate oxygen demand (16), and the expression of genes associated with these conditions may be essential for the ability of cancer cells to adapt to and survive in these microenvironments. Therefore, the metabolic changes that occur in cancer cells may represent a potential therapeutic target for enzymes capable of inhibiting these survival mechanisms. This possibility is relevant for patients with gastric cancer because these patients demonstrate a relatively low sensitivity to conventional chemotherapeutic agents compared to patients with other malignancies. In this study, we first examined the expression of various molecules involved in the human gastric cancer glycolytic pathway and then evaluated a potential therapeutic target by correlating patient prognoses with *in vitro* test results.

We evaluated the expression of principle molecules related to the glycolytic pathway including HIF-1α, which is a transcription factor that targets genes involved in extracellular glucose import, GLUT-1, which is important for glycolysis and the catabolism of intracellular glucose, and HK-2, which functions to inhibit mitochondrial proteins such as PDK-1. The aggressiveness of malignant tumors may be caused by aberrant glucose metabolism induced by HIF-1α because several of the glycolytic enzymes are important regulators of tumor cell death and apoptosis (5,17). Thus, we aimed to verify the relationship between HIF-1α expression and that of other enzymes involved in gastric cancer. The results from our immunohistochemical analysis of human gastric cancer tissues demonstrated that the expression of PDK-1, a gate-keeping enzyme that regulates carbohydrate transport into the mitochondria, was significantly correlated with HIF-1α expression.

Few studies have evaluated the correlation between the expression of enzymes related to glycolysis and gastric cancer prognosis; GLUT-1 is the only protein that has been evaluated (10,11). Kawamura *et al*(10) reported that positive GLUT-1 expression progressively increased with more advanced stages of cancer and was correlated with poor gastric cancer prognosis. However, correlations between the expression of other enzymes, such as HK-2 and PDK-1, and gastric cancer prognosis have not been previously reported. In this study, we evaluated these potential correlations and found that although the expression of GLUT-1 and PDK-1 was frequently observed in samples from patients with more advanced disease, poor prognosis was only associated with PDK-1 expression. With regard to the GLUT-1 results, we assumed that the difference between this study and previous reports was due to differences in the disease stage of the enrolled patients. The percentage of patients who were diagnosed with advanced gastric cancer in our study (72.4%) was higher than that reported in previous studies, which may indicate that PDK-1 can serve as a more specific prognostic marker for more advanced disease stages.

PDK-1 is a critical enzyme for attenuating the production of mitochondrial reactive oxygen species and maintaining ATP levels, and it is also a direct target of HIF-1α. PDK-1 can inactivate the pyruvate dehydrogenase E1α subunit that converts pyruvate to acetyl-CoA, which inhibits pyruvate metabolism via the tricarboxylic acid cycle (18). To date, four PDK-1 isoforms have been verified in human tissue, and the expression of these isoforms was shown to be organ specific (19,20). These PDK-1 isoforms have been detected in the liver, muscle, and pancreas. With regard to malignant cells, several studies have reported that PDK-1, which is targeted by HIF-1α, can control the switch from aerobic oxidation to glycolytic glucose metabolism. This metabolic switch is advantageous to tumor growth because it reduces mitochondrial oxygen consumption, which results in preventing the accumulation of reactive oxygen species (21,22). In this study, changes in the use of metabolic processes such as glucose uptake and lactate production in gastric cancer cell lines were dependent on PDK-1 expression. In addition, we assumed that these metabolic changes were predominant in cancer cell lines because HEK-293, a non-cancerous cell line, demonstrated little metabolic change despite high PDK-1 expression.

In our *in vivo* study, PDK-1 expression correlated with cancer progression and patient prognosis. Moreover, the expression of PDK-1 was more meaningful as a prognostic marker for patients who were administered single-agent 5-FU as adjuvant treatment. Using an *in vitro* test, we confirmed that the responsiveness of MKN45 cells, which expressed a high level of PDK-1, to therapeutic 5-FU was lower than that of AGS cells. A metabolic advantage, such as increased glycolysis or mitochondrial dysfunction, may enhance the aggressiveness of tumors and increase their resistance to chemotherapeutic agents (17). In addition, these changes can negatively regulate the activities of pro-apoptotic molecules such as Bax, Bak and Bad, which may lead to the prevention of cell death triggered by chemotherapeutic agents. In cancer cells, PDK-1 is required for the conversion of pyruvate into lactate rather than acetyl-CoA, which occurs in the first step of the TCA cycle (21,22). As a result, PDK-1 may represent a target for additional treatment options that could be added to conventional therapies.

DCA is a non-specific mitochondrial PDK-1 inhibitor. By blocking the function of this enzyme, DCA decreases lactate production by shifting pyruvate metabolism from glycolysis to oxidative phosphorylation in the mitochondria. This relatively nontoxic and inexpensive small molecule has been clinically administered to treat patients with lactic acidosis caused by a genetic disorder (23). Recently, DCA has been considered as a potential cancer therapeutic agent for several malignant tumors originating from the endometrium, breast and brain (24–26). However, DCA has not yet been considered a therapeutic option for the treatment of gastric cancer. In our *in vitro* experiments, DCA treatment had a similar effect on the viability of gastric cancer and normal kidney cell lines. However, this treatment led to significant metabolic changes in only the MKN45 cancer cell line, where it resulted in increased lactate production and greater PDK-1 expression. In addition, the metabolic changes associated with cancer cells may contribute to an increased sensitivity to chemotherapy and radiation therapy (27,28). Previous research has reported that DCA treatment could induce apoptosis in malignant cells by causing mitochondrial dysfunction and activating the NFAT-Kv1.5 pathway (26,29). Moreover, this induction of apoptosis is also responsible for cancer cell sensitivity to conventional treatment. In this study, we evaluated the therapeutic effects resulting from treatment with DCA, a PDK-1 inhibitor, and the synergic effects of DCA and 5-FU treatment in a gastric cancer cell line expressing a high level of PDK-1 and producing a high level of glycolytic products. This is the first study to demonstrate that low-dose DCA treatment provides a minimal effect on cell survival but causes a metabolic shift in gastric cancer cells expressing PDK-1. Therefore, our results suggest that DCA may represent an additional agent that could be used in combination with conventional chemotherapy for patients with a low responsiveness to therapy and a poor prognosis.

In conclusion, PDK-1 overexpression in cancer tissues was correlated with poor prognosis in patients with gastric cancer. DCA, an inhibitor of PDK-1, led to metabolic changes in gastric cancer and may therefore serve as an additional treatment option for patients with gastric cancers expressing PDK-1 and for those with a poor prognosis.

## Figures and Tables

**Table I.** The characteristics of the 152 patients enrolled in this study.

Characteristics	No.	Percentage (%)
Age		
<65	99	65.1
≥65	53	34.9
Gender		
Male	96	63.2
Female	56	36.8
Approach		
Open	109	71.7
Laparoscopy	43	28.3
Resection		
Total gastrectomy	36	23.7
Subtotal gastrectomy	116	76.4
Reconstruction		
Billroth-I	79	52.0
Billroth-II	39	25.7
Roux en Y	34	22.3
LN dissection		
D1+	68	44.7
D2	84	55.3
Combined resection		
Yes	23	15.1
None	129	84.9
*Helicobacter pylori*		
Infection	132	86.8
Non-infection	20	13.2
Adjuvant treatment		
Yes	97	63.8
None	55	36.2
Location		
Upper	27	17.8
Middle	43	28.3
Lower	82	53.9
Tumor invasion		
T1	42	27.6
T2	31	20.4
T3	37	24.3
T4	42	27.6
LN metastasis		
N0	47	30.9
N1	29	19.1
N2	38	25.0
N3	38	25.0
Histology		
Signet ring/mucinous	46	30.3
Others	106	69.7

LN, lymph node.

**Table II.** Correlation between hypoxia-inducible factor-1α expression and other glycolysis-related enzymes.

	HIF-1α		
N (%)	Negative (n=105) (%)	Positive (n=47) (%)	P-value
GLUT-1			
Negative (n=116)	77 (50.7)	39 (25.7)	0.196
Positive (n=36)	28 (18.4)	8 (5.3)	
HK-2			
Negative (n=145)	99 (65.1)	46 (30.3)	0.437
Positive (n=7)	6 (3.9)	1 (0.7)	
PDK-1			
Negative (n=133)	96 (63.2)	37 (24.3)	0.029
Positive (n=19)	9 (5.9)	10 (6.6)	

HIF-1α, hypoxia-inducible factor-1α; GLUT, glucose transporter; HK, hexokinase; PDK, pyruvate dehydrogenase kinase.

**Table III.** Correlation between GLUT-1, HK-2 and PDK-1 expression and the presence of pathological features.

		GLUT-1			HK-2			PDK-1		
Characteristics	N	Negative (n=116) (%)	Positive (n=36) (%)	P-value	Negative (n=145) (%)	Positive (n=7) (%)	P-value	Negative (n=133) (%)	Positive (n=19) (%)	P-value
Location										
Upper	27	18 (66.7)	9 (33.3)	0.149	25 (92.6)	2 (7.4)	0.226	21 (77.8)	6 (22.2)	0.170
Middle	43	37 (86.0)	6 (14.0)		43 (100.0)	0 (0.0)		40 (90.3)	3 (7.0)	
Lower	82	61 (74.4)	21 (25.6)		77 (93.9)	5 (6.1)		72 (87.8)	10 (12.2)	
T stage										
T1	42	38 (90.5)	4 (9.5)	0.011	40 (95.2)	2 (4.8)	0.955	41 (97.6)	1 (2.4)	0.020
T2/T3/T4	110	78 (70.9)	32 (29.1)		105 (95.5)	5 (4.5)		92 (83.6)	18 (16.4)	
N stage										
N0	47	42 (89.4)	5 (10.6)	0.011	46 (97.9)	1 (2.1)	0.330	45 (95.7)	2 (4.3)	0.040
N1/N2/N3	105	74 (70.5)	31 (29.5)		99 (94.3)	6 (5.7)		88 (83.8)	17 (16.2)	
Size (cm)										
<5	77	63 (81.8)	14 (18.2)	0.106	75 (97.4)	2 (2.6)	0.231	73 (94.8)	4 (5.2)	0.006
≥5	75	53 (70.7)	22 (29.3)		70 (93.3)	5 (6.7)		60 (80.0)	15 (20.0)	
Lauren										
Diffuse	62	55 (88.7)	7 (11.3)	0.016	59 (95.2)	3 (4.8)	0.106	52 (83.9)	10 (16.1)	0.564
Intestinal	58	37 (63.8)	21 (36.2)		57 (98.3)	1 (1.7)		53 (91.4)	5 (8.6)	
Mixed	21	16 (76.2)	5 (23.8)		18 (85.7)	3 (14.3)		19 (90.5)	2 (9.5)	
Unknown	11	8 (72.7)	3 (27.3)		11 (100.0)	0 (0.0)		9 (81.8)	2 (18.2)	
Histologic type										
SRC/mucinous	46	40 (87.0)	6 (13.0)	0.042	44 (95.7)	2 (4.7)	0.921	38 (82.6)	8 (17.4)	0.230
Others	106	76 (71.7)	30 (28.3)		101 (95.3)	2 (4.3)		95 (89.6)	11 (10.4)	
*Helicobacter pylori*										
Yes	132	102 (77.3)	30 (22.7)	0.476	127 (96.2)	5 (3.8)	0.217	114 (86.4)	18 (13.6)	0.276
No	20	14 (70.0)	6 (30.0)		18 (90.0)	2 (10.0)		19 (95.0)	1 (5.0)	

GLUT, glucose transporter; HK, hexokinase; PDK, pyruvate dehydrogenase kinase; SRC, signet ring cell.

**Table IV.** The disease-free and overall survival rates in relation to the clinicopathological features of the patients.

		Disease-free survival			Overall survival		
Characters	N	Mean survival (month)	95% CI	P-value	Mean survival (month)	95% CI	P-value
Age							
<65	99	31.7	29.6–33.9	0.790	51.0	48.4–53.7	0.397
≥65	53	32.2	29.0–35.3		49.0	44.8–53.2	
Gender							
Male	96	32.4	30.1–34.7	0.956	47.9	44.8–51.0	0.070
Female	56	31.7	28.8–34.6		53.1	50.3–55.9	
Size (cm)							
<5	77	34.6	32.5–36.7	0.012	50.1	48.5–52.7	0.007
≥5	75	29.5	26.7–32.3		47.2	43.3–51.1	
Tumor location							
Upper	27	26.9	22.2–31.5	0.040	47.9	42.1–53.6	0.819
Middle	43	33.5	30.5–36.4		52.1	48.4–55.8	
Lower	82	32.9	30.5–35.3		50.0	46.8–53.2	
Tumor invasion							
T1/T2/T3	110	33.5	31.7–35.2	<0.001	51.9	49.5–54.2	0.023
T4	42	27.6	23.4–31.7		46.2	40.9–51.6	
Lymph node							
N0/N1	76	36.9	35.6–38.1	<0.001	54.4	52.6–56.2	<0.001
N2/N3	76	27.3	24.3–30.3		43.9	40.2–47.7	
Adjuvant							
No	55	34.4	32.2–36.6	0.015	49.0	44.8–49.2	0.026
Yes	97	30.8	28.3–33.3		48.4	45.2–51.6	
Histologic type							
Differentiated	49	35.00	32.5–37.5	0.052	43.9	40.7–47.1	0.701
Undifferentiated	103	30.48	28.2–32.8		50.1	47.3–52.9	
*Helicobacter pylori*							
Negative	20	29.1	23.0–35.1	0.190	49.1	42.8–55.3	0.880
Positive	132	32.1	30.3–33.9		50.4	48.0–52.8	
HIF-1α							
Negative	105	32.4	30.2–34.6	0.918	49.9	47.0–52.7	0.464
Positive	47	31.0	28.0–34.0		50.5	46.8–54.3	
GLUT-1							
Negative	116	32.3	30.5–34.2	0.255	50.9	48.5–53.4	0.502
Positive	36	30.2	25.7–34.6		41.8	37.3–46.4	
HK-2							
Negative	145	32.4	30.5–34.2	0.780	50.7	48.4–52.9	0.201
Positive	7	27.8	22.2–33.5		37.1	25.4–48.9	
PDK-1							
Negative	133	33.1	31.3–35.0	0.037	51.3	49.1–53.6	0.015
Positive	19	26.3	20.3–32.3		38.1	31.2–44.9	

CI, confidential interval; HIF, hypoxia-inducible factor; GLUT, glucose transporter; HK, hexokinase; PDK, pyruvate dehydrogenase kinase.

**Table V.** The disease-free and overall survival rates in 78 patients who were administered 5-fluorouracil as adjuvant treatment.

		Disease-free survival			Overall survival		
Characters	N	Mean survival (month)	95% CI	P-value	Mean survival (month)	95% CI	P-value
Age							
<65	43	30.5	27.2–33.8	0.621	50.4	46.2–54.7	0.313
≥65	35	31.0	26.9–35.0		46.8	41.2–52.5	
Gender							
Male	49	30.4	26.8–34.0	0.314	45.3	40.4–50.1	0.048
Female	29	32.7	29.1–36.3		53.5	50.1–56.9	
Size (cm)							
<5	29	31.7	27.2–36.3	0.733	48.3	43.9–52.7	0.421
≥5	49	30.8	27.6–34.0		47.6	42.9–52.3	
Tumor location							
Upper	17	27.0	20.9–33.1	0.328	48.0	40.7–55.4	0.986
Middle	16	32.1	27.0–37.2		47.7	39.2–56.2	
Lower	45	32.1	28.8–35.4		49.2	44.9–53.5	
Tumor invasion							
T1/T2/T3	54	32.0	29.1–34.9	0.162	50.3	46.6–54.0	0.236
T4	24	29.0	23.7–34.3		45.5	38.1–52.9	
Lymph node							
N0/N1	29	35.9	33.1–38.7	0.014	51.7	47.1–56.3	0.172
N2/N3	49	27.8	24.3–31.2		44.7	40.3–49.1	
Histologic type							
Differentiated	23	31.6	26.6–36.6	0.938	41.2	35.7–46.6	0.609
Undifferentiated	55	30.8	27.8–33.9		49.4	45.4–53.4	
*Helicobacter pylori*							
Negative	11	29.5	21.1–37.8	0.670	50.7	42.7–58.7	0.429
Positive	67	31.2	28.5–33.9		48.3	44.5–52.1	
HIF-1α							
Negative	56	31.8	28.6–34.9	0.599	49.8	46.0–53.5	0.453
Positive	22	29.4	24.6–34.1		45.4	37.9–52.9	
GLUT-1							
Negative	59	31.6	28.4–34.7	0.818	49.0	45.0–53.0	0.738
Positive	19	30.2	25.6–34.8		45.9	39.6–52.2	
HK-2							
Negative	75	Not evaluable		0.361	Not evaluable		0.433
Positive	3						
PDK-1							
Negative	65	32.8	30.1–35.6	0.026	51.1	47.9–54.3	0.002
Positive	13	23.8	16.7–30.8		33.3	24.3–42.3	

CI, confidential interval; HIF, hypoxia-inducible factor; GLUT, glucose transporter; HK, hexokinase; PDK, pyruvate dehydrogenase kinase.

**Table VI.** Factors predicting prognosis in the multivariable analysis.

	All patients				Patients using single-agent 5-flurouracil adjuvant treatment			
Characters	P-value	β	OR	95% CI	P-value	β	OR	95% CI
Disease-free survival								
Lymph node								
N0/N1 vs. N2/N3	<0.001	2.186	8.897	2.640–29.986	0.015	1.831	6.238	1.418–27.438
PDK-1								
Negative vs. positive					0.010	1.311	3.709	1.377–9.991
Overall survival								
Lymph node								
N0/N1 vs. N2/N3	0.003	1.828	6.224	1.826–21.219	0.049	1.440	4.220	1.005–17.725
PDK-1								
Negative vs. positive	0.030	1.061	2.890	1.108–7.536	0.004	1.635	5.132	1.699–15.503

OR, odds ratio; CI, confidential interval; PDK, pyruvate dehydrogenase kinase.
